# Neural Changes after Vision Therapy in Convergence Insufficiency: A Systematic Review

**DOI:** 10.1155/2024/5586202

**Published:** 2024-07-30

**Authors:** Laura Barberán-Bernardos, Natalia Jiménez, David P. Piñero

**Affiliations:** ^1^ Department of Optics, Pharmacology and Anatomy University of Alicante, Alicante, Spain; ^2^ Department of Ophthalmology (IMQO-Oftalmar) Vithas Medimar International Hospital, Alicante, Spain

## Abstract

**Objective:**

To investigate if the use of vision therapy (VT) in convergence insufficiency (CI) has a significant neural impact and how it correlates with the clinical changes occurring with this option of treatment.

**Methods:**

A systematic review of the scientific literature was carried out in the PubMed and Scopus databases, where all the scientific literature on the neural impact of VT in CI was analyzed. A total of 17 articles were initially found and a detailed analysis was carried out. After full-text reading, only four studies met the defined inclusion criteria. The following data from them were extracted: CI cases and controls, clinical and neural parameters evaluated, the neural response to VT observed, type of study, and VT performed. The quality of the studies was assessed using the GRADE tool.

**Results:**

Some neural changes have been reported after VT in CI with the use of functional magnetic resonance imaging (fMRI). Specifically, a modification of the functional activity of some brain areas (especially front fields, oculomotor vermis, and cerebellum) was found. However, contradictory findings in terms of the change in functional activity (increase or decrease) were found that might be associated to differences in fMRI protocols. In the GRADE analysis, serious concerns were found in the categories of risk of bias, inconsistency, indirectness, and imprecision, so the certainty of evidence for each outcome was very low.

**Conclusion:**

The research performed to this date does not allow confirming if there are neural changes occurring after vision therapy in patients with CI because the quality of the research performed on this issue is very low, with several methodological concerns.

## 1. Introduction

Convergence insufficiency (CI) is the most common binocular disorder, with a variable prevalence depending on the population evaluated (2.25–33%) [1, 2]. Patients with CI cannot properly maintain the ocular alignment and focus at near distance [3, 4], which causes high levels of symptomatology and hinders the patient's quality of life [5]. The most common symptoms are diplopia, headaches, eye strain, lack of concentration, sleepiness at near vision activities, blurry near vision, floating or mixed words when reading, and even eye pain [6, 7]. The Convergence Insufficiency Symptom Survey (CISS) is a validated instrument widely used to evaluate these symptoms and to detect CI [8]. Besides symptoms, there are several clinical signs associated to the presence of CI, including greater exophoria at near than at distance (difference >4∆), a receded near point of convergence (NPC), reduced positive fusional vergences (PFV) in near vision (≤15∆), a reduced accommodative convergence to accommodation ratio (AC/A), and reduced binocular accommodative facility (BAF), with difficulty in focusing through positive lenses (≤3 cycles per minute) [9, 10].

Concerning the treatment of CI, accommodative and vergence therapy has been demonstrated to be an effective method to alleviate the symptoms while eliminating the altered signs [9, 11, 12]. Specifically, office-based therapy has been proven as even more efficient than home-based therapy [9]. Prisms are also sometimes used but they are inefficient in children [13].

The symptoms and clinical diagnosis of this condition have been widely studied, but its neural implications are less known. Specifically, knowledge on the correlation between neural and clinical changes in CI is limited. Indeed, there are few studies on how CI affects the functional activity and neural areas. Some neural changes seem to occur in patients with CI after vision therapy which are characterized by parameters such as spatial extent or BOLD signal within several brain areas, including the frontal, temporal, parietal, and occipital lobes and the cerebellum [14–17]. Learning about the impact of vision therapy techniques on diverse neural parameters can allow the clinician to optimize these procedures and therefore maximize the results obtained with them.

The objective of this systematic review was to investigate if vision therapy in CI has a significant neural impact and how it correlates with the clinical changes occurring with this option of treatment.

## 2. Methods

In this study, a search was performed in PubMed and Scopus databases. The goal of this systematic review was to confirm if vision therapy in CI has a significant impact on neural activity characterized by functional magnetic resonance. The search equation used in both platforms was the following: *“convergence insufficiency” AND (neural OR “magnetic resonance”) AND therapy.* The list of references obtained with the search was analyzed comprehensively and only the articles meeting the following inclusion criteria were considered: (i) clinical trials and pseudo-experimental studies on neural changes in CI with any modality of visual training, (ii) studies conducted in humans without neurological pathology, regardless of age and sex, (iii) studies published in English or Spanish, and (iv) studies including an analysis of neural modifications after visual training using functional magnetic resonance imaging (fMRI). Studies about neural changes in CI associated to neurological alterations, such as neurodegenerative diseases, neurodevelopmental disorders, or brain damage, were excluded.

After the search was performed, duplicates were removed. Then, a title and abstract review of the remaining articles was conducted first, selecting the articles that met the inclusion criteria of this review. Finally, a full-text review of these articles was conducted, extracting the following data from them: author, publication year, number of participants, number of cases with CI and controls, sex, clinical parameters evaluated, neural parameters evaluated, the neural response to vision therapy observed, type of study, and vision therapy performed.

After this, a quality assessment of the articles was conducted using the Grading of Recommendations Assessment, Development, and Evaluation (GRADE) tool, considering the following factors: study design, risk of bias, indirectness, inconsistency, imprecision, and publication bias [18]. Specifically, the risk of bias was evaluated using the Risk of Bias in Non-Randomized Studies-of Interventions (ROBINS-I) and the revised Cochrane risk-of-bias tool for randomized trials (RoB 2) for randomized trials, respectively [19, 20]. The overall quality of the evidence was rated as high, moderate, low, or very low following the guidelines of the GRADE tool [21].

## 3. Results

**Figure 1:**
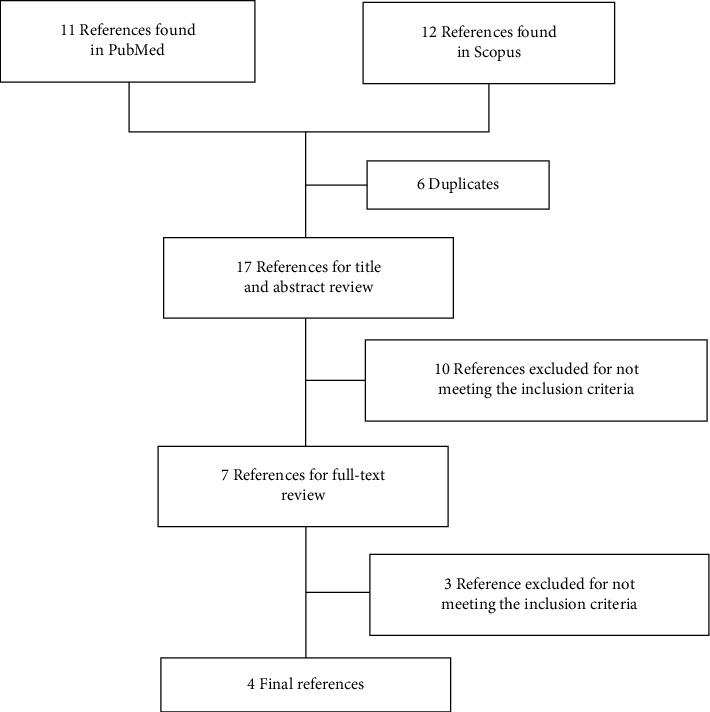
Flowchart showing the search process in the current systematic review.

On the initial search, 11 articles were found in PubMed and 12 in Scopus (Figure 1). After removing duplicates, a total of 17 articles were obtained and their title and abstract were comprehensively analyzed. After this evaluation, ten additional articles were excluded as they were not meeting the inclusion criteria of this review. Then, a full-text review of the remaining 7 articles was performed. Three of these articles were also excluded (all of them for not being clinical trials nor pseudo-experimental pre-post studies) and only four articles were finally selected [15–17, 22].

The most important data (author and year, characteristics of the sample, clinical and neural parameters measured, type of study, and how the vision therapy was performed) of the selected studies are summarized in Table 1. These four studies analyzed a total of 84 patients, of whom 52 presented CI and 32 presented normal binocular vision. The age range among CI patients ranged from 18 to 35 years.

The most recent study by Alvarez et al. [17] presented the largest number of participants, both CI and controls, and an equitable distribution in terms of sex. All the studies included the evaluation of the NPC, PFV, the magnitude of the phoria, and symptomatology with the CISS questionnaire. Furthermore, most of studies evaluated the neural response in the front fields, oculomotor vermis, and the cerebellum [16, 17, 22]. Three of the studies compared a sample of CI subjects with binocular normal subjects [16, 17, 22]. Only the study by Widmer et al. [15] used a sample consisting entirely of CI subjects, but the authors administrated a placebo vision therapy to half of the sample.

Table 2 shows the clinical alterations of CI before vision therapy. Headaches, eye strain, and blurred vision were the most reported symptoms in the studies revised [16, 17]. Furthermore, NPC, PFV, and CISS score were clinical parameters evaluated in all the studies, with similar results in all of them that were indicative of CI [15–17, 22]. The persistent presence of symptoms and the altered results of the clinical parameters previously mentioned in the different studies showed consistency in the clinical manifestation of untreated CI. The magnitude of the phoria was also measured by Alvarez et al. resulting in an exodeviation of 4 ∆ greater at near than at distance [17]. This difference in the phoria is related to the difficulties to maintain a proper convergence focusing on near objects. In most of studies, stereopsis was only considered as an inclusion or exclusion parameter, but with careful analysis of its changes. Ocular movement analysis revealed the presence of alterations on the coordination and smoothness of the eyes during convergence [22].

Table 3 shows the neural findings before vision therapy of the CI subjects. A decrease in BOLD signal, spatial extent, and functional activity compared to binocular normal subjects was found in several regions of interest, including the frontal, temporal, parietal, and occipital lobes and the cerebellum [16, 17, 22]. The study by Widmer et al. found several areas of activation within the cerebellum and the frontal, temporal, parietal, and occipital lobes [15].

The clinical parameters analyzed after vision therapy are listed in Table 4. Several binocular parameters, such as NPC, PFV, and the level of exophoria at near, showed an improvement [15–17, 22]. Specifically, there were an increase of the vergence capacity (>15∆) after the use of vision therapy in CI. Alvarez et al. also confirmed the development of a faster response of eye movements [22]. A decrease of the symptoms reported by the participants was observed by using the CISS [15–17, 22].

Table 5 shows a summary of the neural parameter after vision therapy in CI cases. Three of the studies analyzed found an increased functional activity of several brain areas when comparing pre- and post-therapy [16, 17, 22]. However, Widmer et al. found that after therapy, some of these areas showed a decreased functional activity [15].

Finally, a quality assessment of the articles retrieved was performed using the GRADE tool for each different outcome (neural changes within the frontal, parietal, and occipital lobes, the cerebellum, and the brainstem), as shown in Table 6. The risk of bias was assessed with the ROBINS-I tool for the three non-randomized studies and the RoB 2 for the only randomized trial retrieved (Table 7), obtaining serious or high risk of bias in all cases. In the GRADE analysis, serious concerns were found in the categories of risk of bias, inconsistency, indirectness, and imprecision, so the certainty of evidence for each outcome was very low. Specifically, the concerns in inconsistency resided in the heterogeneous and contradictory results. The low results in the category of indirectness were due to the different methodology used in each study as well as to the differences in the stimuli used to analyze the neural response and differences in the populations evaluated. For the imprecision domain, the follow-up periods of the studies were too short and unsuitable for characterizing an effect on the long term and the samples were too small and unrepresentative in most of the studies.

## 4. Discussion

There is a limited body of literature addressing the neurological implications of convergence insufficiency. The visual system is extremely complex, and the study of neural changes often demands the use of techniques and research approaches that may not always be widely accessible in all instances. In fact, the implication of a multidisciplinary team with medical professionals from areas such as neurology or radiology is necessary in this research area.

Eyes with CI have difficulty in coordinating and converging effectively towards a near point, but various methods, including the utilization of prisms and lenses, can be implemented to address this difficulty. However, the most impactful and successful approach is vision therapy [23]. At present, vision therapy stands out as the foremost treatment prescribed in clinical settings, often combined with a 12-week regimen of home exercises [9]. During the assessment of changes in standard clinical parameters, a decrease in breakage and recovery of NPC, along with an increase in PFV, and an improvement in near exophoria have been observed and reported by previous authors, including those analyzed in the current systematic review [7, 9, 12, 13]. Moreover, the subjects evaluated in the four studies revised underwent anatomical fMRI tests to rule out the presence of lesions or disorders. The findings of these analyses suggested that vision therapy for CI might induce alterations in both clinical metrics and functional neural changes, demonstrating an augmented functional activity in specific regions. The reported neural changes were in the cerebellum, the front fields, and the oculomotor vermis [16, 17, 22].

Using vision therapy in subjects with CI helped to improve NPC and PFV, increase the mean peak velocity convergence, and increase the functional activity in the frontal lobe, cerebellum, and brainstem. A correlation between the clinical and the cortical signs was also found. Therefore, treating CI through vision therapy seems to involve neural changes within the plasticity of the neural and muscular system.

Three of the four studies in this systematic review were carried out by professionals of the same group of investigation and performed vision therapy in a sample of subjects with CI and in binocular normal subjects [16, 17, 22]. Therefore, the same methodology was used in these studies and the results obtained were like each other. As previously mentioned, the fourth study employed a sample entirely of subjects with CI but performing in a subgroup a placebo therapy and in another one a binocular-accommodation vision therapy [15]. This study found different outcomes, which may be probably explained by different factors, including differences in the type of stimuli used while obtaining the fMRI images. The study by Widmer et al. used a random-dot stereogram stimulus at a fixed distance, presenting a disparity of 480 seconds [15], and although the three remaining studies used two different stimuli, both were associated with vergence demands: LED target with a disparity of 2°, 3°, and 4° in the case of Alvarez et al. and two eccentric squares with a disparity of 4° and 6° in the case of Alvarez et al. [16, 17, 22]. It should be considered that the type of cortex activation analyzed by functional magnetic resonance differs when using a 3D stimuli (such as a stereogram) versus a 2D stimuli [24], which may explain some of the differences found between the studies analyzed in the type of neural response after vision therapy. This highlights the need for a standardized evaluation of the neural response in studies characterizing the impact of vision therapy in brain activity. Besides the type of stimuli, there may be more methodological differences that may have contributed to the variability of the results from the different studies revised in the current systematic review, such as differences in the population age or the severity of CI [16, 17, 22].

Widmer et al. [15] found a decrease in functional activity in the frontal lobe (dorsolateral prefrontal cortex and frontal eye fields), parietal lobe (inferior parietal lobule), occipital lobe (precuneus), and cerebellum (cerebellar vermis) after performing vision therapy only in subjects with CI. Specifically, patients with CI who underwent placebo therapy (consisting of tasks with no vergence or accommodation demand) did not show this neural effect. The three remaining studies found an increased functional activity of some of these areas after vision therapy in subjects with CI [16, 17, 22]. This result suggests that vision therapy might have some impact on brain function, leading to an improvement of the coordination between the eye muscles and consequently of ocular convergence. This heightened functional activity may play a role in enhancing the visual function and may have the potential to positively influence on the execution of daily visual tasks and overall visual performance. However, more studies are needed to define the exact relationship between neural changes in CI after vision therapy and the clinical improvements observed with this therapeutic approach.

In terms of the quality of the studies, the analysis with the GRADE tool revealed that the level of quality of the studies revised was very low mainly due to the sample size limitations, the inadequate selection of the control group (no inclusion of CI groups with no treatment or placebo treatment in three studies), and the different protocols used to evaluate the neural response. Only the most recent study [17] included an acceptable number of subjects in the CI and control groups, but controls were patients with normal binocular vision and consequently without any type of visual treatment. Besides this, the follow-up was extremely short in most of studies, not allowing us to confirm if the neural changes observed with vision therapy were maintained over time. In any case, it should be considered that there are some limitations to perform this type of studies, such as the difficulty in recruiting patients, the lack of consensus on the definition of the fMRI protocol to follow to better evaluate the neural response to visual exercises, and funding restrictions. It should be noted here that fMRI, despite being a non-invasive test, requires complex medical procedures and a technical and specialized team. The costs of this technology are usually high, depending on the duration of the scanning, the quantity of image sequences, or the need for contrast.

In conclusion, the studies performed to this date do not allow confirming if there are neural changes occurring after vision therapy in patients with CI because the quality of the research performed on this issue is very low, with several methodological concerns. Indeed, some contradictory findings have been reported in the neural response associated to vision therapy in terms of functional activity (increase or decrease). Future studies should be conducted on this issue with an adequate selection of the number of patients (sample size calculation) and of the type of control group. In addition, there is a need for studies with larger samples (multicentric studies would be very helpful) using standardized protocols of visual stimulation during fMRI evaluation and evaluating the impact on a longer term to confirm the stability of the neural modifications induced.

## Figures and Tables

**Table 1:** Main results of the studies included in this systematic review.

Author (year)	Sample (*N*)	Age (years)	Sex	Study design	Clinical parameters	Neural parameters	Neural response measurement	Vision therapy
Alvarez et al. (2019)	CI *n* = 25	20.3 ± 3.2	10M 15F	Pseudo-experimental study	NPC PFV Phoria CISS	BOLD signal Mean beta weights	Functional activity of FEF, SEF, PEF and oculomotor vermis	12 h OBVAT
	BNC *n* = 25	21.3 ± 3.1	14M 11F					

Widmer et al. (2018)	CI *n* = 7	26.1 ± 2.5	1M 6F	Clinical trial	NPC PFV, NFV Phoria CISS AA AF, VF	BOLD signal fMRI	(i) FEF, SEF (ii) Precuneus (iii) Cerebellar vermis (iv) DLPFC (v) IPL	(i) 12 weeks OBVAT (*n* = 4) (ii) Placebo therapy (*n* = 3)

Jaswal et al. (2014)	CI *n* = 7	(18–35)	4F	Pseudo-experimental study	NPC PFV Phoria CISS	fMRI BOLD signal	(i) FEF (ii) PPC (iii) Cerebellar vermis	6 h HBT 12 h OBVAT
	BNC *n* = 7	(18–35)	4M 3F					

Alvarez et al. (2010)	CI *n* = 13	23 ± 1.7 (20–26)	4F	Pseudo-experimental study	NPC PRC PFV Phoria CISS	fMRI BOLD signal General linear model	(i) DLPFC (ii) FEF, SEF, PEF (iii) Cerebellum (iv) Brain stem	18 h
	BNC *n* = 13	25 ± 4 (21–35)	9M 4F					

CI, convergence insufficiency; BNC, binocular normal control; *M*, male; F, female; NPC, near point of convergence; PFV, positive fusional vergence; NFV, negative fusional vergence; CISS, Convergence Insufficiency Symptom Survey; fMRI, functional magnetic resonance imaging; FEF, frontal eye field; PEF, parietal eye field; SEF, supplementary eye field; OBVAT, office-based vergence/accommodative therapy; AA, amplitude of accommodation; AF, accommodative facility; VF, vergence facility; IPL, inferior parietal lobule; HBT, home-based therapy; BOLD, blood oxygenation level dependent; DLPFC, dorsolateral prefrontal cortex; PPC, posterior parietal cortex; PRC, recovery point of convergence.

**Table 2:** Clinical parameters in patients with CI before vision therapy.

Author (year)	*N*	Symptoms	NPC (cm)	PFV	Phoria	CISS (points)	Stereopsis	Eye movements
Alvarez et al. (2019)	25	(i) Headaches (ii) Eye strain (iii) Blurred vision (iv) Diplopia	≥6	Reduced ≤15∆ at near	4∆ greater exo at near than at distance	≥21	Normal	Not evaluated

Widmer et al. (2018)	7	—	8 ± 1.5	13.4 ± 1.5 ∆	10 ± 4.3 ∆ exophoria	32.6 ± 9.5	Better than 500 sec arc	Not evaluated

Jaswal et al. (2014)	4	(i) Headaches (ii) Eye strain (iii) Blurred vision (iv) Slow reading	≥6	Reduced ≤15∆ at near	Not provided	≥21	Normal	Not evaluated

Alvarez et al. (2010)	4	(i) Asthenopic complaints (ii) Eye strain	≥6	Reduced ≤15∆ at near	Not provided	≥21	Better than 70 sec arc	Slow convergence response

NPC, near point of convergence; PFV, positive fusional vergence; CISS, Convergence Insufficiency Symptom Survey.

**Table 3:** Neural findings in patients with CI before vision therapy.

Author (year)	*N*	Frontal lobe	Temporal lobe	Parietal lobe	Occipital lobe	Cerebellum
Alvarez et al. (2019)	25	Reduced spatial extent and peak functional activity within the FEF and SEF compared to BNC	Not provided	Reduced spatial extent and peak functional activity within the PEF compared to BNC	Not provided	Reduced spatial extent and peak functional activity within the oculomotor vermis compared to BNC
Widmer et al. (2018)	7	Activation in the inferior frontal gyrus, precentral gyrus, medial frontal gyrus, and superior frontal gyrus	Activation in the superior temporal gyrus and middle frontal gyrus	Activation in inferior parietal lobule	Activation in the occipital lobe	Activation in the posterior lobe/declive
Jaswal et al. (2014)	4	Decreased BOLD signal within the FEF compared to BNC	Not provided	Decreased BOLD signal within the posterior parietal cortex compared to BNC	Not evaluated	Decreased BOLD signal within the cerebellar vermis compared to BNC
Alvarez et al. (2010)	4	Not provided	Not provided	Not provided	Not evaluated	Not provided

BNC, binocular normal control; FEF, frontal eye field; SEF, supplementary eye field; PEF, parietal eye field; BOLD, blood oxygenation level dependent.

**Table 4:** Clinical parameters in patients with CI after vision therapy.

Author (year)	*N*	NPC	PFV	Phoria	CISS	Eye movements
Alvarez et al. (2019)	25	Improved	Increased	Decreased	Improved	Not evaluated
Widmer et al. (2018)	7	Improved	Increased	Not provided	Improved	Not evaluated
Jaswal et al. (2014)	4	Improved	Increased	Improved near dissociated phoria	Improved	Not evaluated
Alvarez et al. (2010)	4	Improved (<6 cm)	Increased (>15Δ)	Decreased	Improved (<16)	Eye speed increased

NPC, near point of convergence; PFV, positive fusional vergence; CISS, Convergence Insufficiency Symptom Survey.

**Table 5:** Neural findings in patients with CI after vision therapy.

Author (year)	*N*	Frontal lobe	Parietal lobe	Occipital lobe	Cerebellum	Brainstem
Alvarez et al. (2019)	25	Increased functional activity within the FEF	Not provided	Not provided	Increased functional activity within the oculomotor vermis	Not provided
Widmer et al. (2018)	7	Decreased activation within dorsolateral prefrontal cortex and FEF. Increased activation in SEF and medial frontal gyrus	Greater activation in the angular gyrus. Decreased activation in inferior parietal lobule	Activation in new areas in the inferior portions. Greater activation in the lingual gyrus, cuneus, and posterior cingulate gyrus. Decreased activation within the precuneus	Decreased activation within cerebellar vermis	Not provided
Jaswal et al. (2014)	4	Increased BOLD signal within the FEF	Increased BOLD signal within the posterior parietal cortex	Not evaluated	Increased BOLD signal within the cerebellar vermis	Not provided
Alvarez et al. (2010)	4	Increased functional activity and spatial extent within the dorsolateral prefrontal cortex, FEF, SEF, and medial frontal gyrus	Average correlation of the parietal area significantly increased in the ROI containing the PEF, precuneus, and Brodmann areas	Not evaluated	Increased functional activity and spatial extent	Increased functional activity and spatial extent

FEF, frontal eye field; PEF, parietal eye field; SEF, supplementary eye field; BOLD, blood oxygenation level dependent.

**Table 6:** GRADE evidence profile.

Quality assessment No. of studies (design)	Risk of bias	Inconsistency	Indirectness	Imprecision	Other considerations	Summary of findings Quality
Neural changes after VT within the frontal lobe						⊕◯◯◯ Very low
4 (1 RCT and 3 PEPP)	Serious	Serious	Serious	Serious	None	

Neural changes after VT within the parietal lobe						⊕◯◯◯ Very low
3 (1 RCT and 2 PEPP)	Serious	Serious	Serious	Serious	None	

Neural changes after VT within the occipital lobe						⊕◯◯◯ Very low
1 (RCT)	High	Low	Low	Serious	None	

Neural changes after VT within the cerebellum						⊕◯◯◯ Very low
4 (1 RCT and 3 PEPP)	Serious	Serious	Serious	Serious	None	

Neural changes after VT within the brainstem						⊕◯◯◯ Very low
1 PEPP	Serious	Low	Low	Serious	None	

GRADE, Grading of Recommendations Assessment, Development, and Evaluation; RCT, randomized controlled trials; PEPP, pseudo-experimental pre-post.

**Table 7:** Methodological quality outcomes using the ROBINS-I tool and the RoB 2.

ROBINS-I	Bias of confounding	Bias in selection of participants into the study	Bias in classification of interventions	Bias due to deviations from intended interventions	Bias due to missing data	Bias in measurement of outcomes	Bias in selection of the reported result	Overall bias
Alvarez et al. (2019)	Low	Low	Serious	Low	Low	Serious	Moderate	Serious
Jaswal et al. (2014)	Low	Low	Serious	Low	Low	Serious	Moderate	Serious
Alvarez et al. (2010)	Low	Low	Serious	Low	Low	Serious	Moderate	Serious

RoB 2	Bias arising from the randomization process	Bias due to deviations from the intended interventions	Bias due to missing outcome data		Bias in measurement of the outcome	Bias in selection of the reported result	Overall bias	Bias in measurement of the outcome

Widmer et al. (2018)	Some concerns	Some concerns	Low		Low	High	High	Low

## Data Availability

Data are available upon reasonable request.
